# Facility characteristics preferred by older men seeking medical male circumcision services in Kenya: qualitative findings from the ‘Tasco’ study (May 2014-June 2016)

**DOI:** 10.1186/s12889-024-19234-x

**Published:** 2024-06-27

**Authors:** Dickens S. Omondi Aduda, Kawango Agot, Spala Ohaga, Appolonia Aoko, Jacob Onyango, Cathy Toroitich-Ruto, Caroline Kambona, Elijah Odoyo-June

**Affiliations:** 1 Jaramogi Oginga Odinga University of Science and Technology, Bondo, Kenya; 2 Impact Research and Development Organization, Kisumu, Kenya; 3 Division of Global HIV & TB, Division of Global Health, US Centers for Disease Control and Prevention, Nairobi, Kenya

**Keywords:** VMMC, Older men, Motivation, Health facilities, Gendered health access

## Abstract

Primary healthcare facilities are central to the implementation of voluntary medical male circumcision (VMMC) as points of access to integrated health services in line with the Kenya AIDS Strategic Framework II (2020/21-2024/25). Knowledge of factors that explain men’s uptake of VMMC and sexual health services at these facilities and preferences of where to get the services remain poorly understood. Using qualitative methodologies, we examined factors that determined facility choice for VMMC services and reasons for preferring the facility among men aged 25–39 years who previously underwent VMMC. The current study draws from focus group discussion interviews with circumcised men and their partners conducted as part of a randomized controlled trial to assess impact of two demand creation interventions in western Kenya. This involved 12 focus group discussions (FGD) with 6–10 participants each. Six FGDs were conducted with circumcised men, and 6 with their sex partners. Thematic issues relevant to a predetermined framework were identified. The themes were organized as follows: service availability, accessibility, affordability, appropriateness and, acceptability. Facility location, physical layout, organization of patient flow, infrastructure, and service provider skills were the outstanding factors affecting the choice of VMMC service outlets by men aged 25–39 years. Additionally, preferences were influenced by individual’s disposition, attitudes, knowledge of VMMC services and tacit balance between their own recognized health needs versus desire to conform to social-cultural norms. Facility choice and individual preference are intricate issues, simultaneously involving multiple but largely intra-personal and facility-level factors. The intrapersonal dimensions elicited may also reflect differential responses to strategic communications and demand creation messages with promotion and prevention frames.

## Introduction

Eastern and southern Africa account for 54% of 38 million people living with HIV worldwide. Approximately 40–60% of new HIV infections reported in this region occur among older adults aged 30 + years, 38–63% among women aged 15–24 years and 14–29% among those aged 15–19 years [1]. A nationally representative survey conducted in Kenya in 2018 showed that HIV burden had declined from 10% in the 1990’s to 4.5% with wide variations across countries ranging from a high of 21.1% in Homabay to 0.2% in Mandera. New cases declined from 75,000 in 2010 to about 41, 416 in 2020 (accounting for 44% against 75% envisaged by 2020) but remains comparatively high. Regions with higher HIV burden (prevalence and new infections) simultaneously also, host traditionally non-circumcising ethnic groups. The new infections occurring among age 15–34 year old males account for 53% of all cases nationally. Despite this progress, there are concerns that the progress has plateaued with corresponding high cases on new HIV infections while awareness of HIV status among people living with HIV remains relatively low. Also, new HIV cases are still more commonly reported in traditionally non-circumcising counties. Additionally, VMMC coverage is lower than 60% against the 2020 target. The Kenya AIDS Strategic Framework II, 2020/21-2024/25 has prioritized scaling up VMMC as a high impact intervention within the HIV programming towards accelerating progress in ending AIDS as a public health threat by the year 2030.

With the scaling up of combination HIV interventions comprising HIV care and treatment, male circumcision, behavior change, and others, the mean annual incidence of HIV infections has consistently declined across contexts. However, the observed changes show varying patterns across geographic and demographic groups over time [1–3]. The emerging patterns of the epidemic and associated inequalities underscores the need to continually improve efficiency and effectiveness of HIV intervention programs.

In 2007 the world health organization (WHO) and the Joint United Nations Program on HIV/ AIDS (UNAIDS) recommended voluntary medical male circumcision (VMMC) as a component of HIV prevention packages in countries with high prevalence of heterosexually-acquired [4]. Kenya is one of the 15 priority countries in eastern and southern Africa that were prioritized by WHO for urgent and rapid VMMC program scale-up [5, 6]. Kenya, was an early adopter having launched in 2008, the National Program of Voluntary Medical Male Circumcision. The program roll out initially focused on the western parts of the country among the *Luos*, a community with substantially higher HIV prevalence (15.3%) than the national estimate of 4.9% and one that does not traditionally practice circumcision [7]. The Kenya VMMC program was very successful during its initial phase and achieved nearly 80% coverage of men aged 15–29 years [8] and is currently transitioning to a sustainability phase where services are more routine and integrated with an essential package of health interventions that includes male sexual and reproductive health. However, by 2015, the proportion of circumcised men aged > 30 years was only about 5% compared to 48% of 10–14 years, which highlighted probable access and demand barriers [9]. There was need to identify effective strategies to address potential barriers to VMMC access and uptake and enhance circumcision coverage among populations with historically lower coverage, such as males aged 25 years and older [10]. Modelling studies have shown that circumcising men ages 20–29 years is cost-saving and critical for achieving immediate impact on increasing the number of HIV infections averted [11]. Addressing these barriers could help to strengthen particularly information and service delivery program components for sustainability strategies.

The Kenya national strategic plan aimed to achieve at least 95% coverage of eligible males with VMMC services by end of 2019 while simultaneously transitioning to the sustainability phase [9]. The role of primary healthcare facilities, the majority of which are government owned, is central to achieving these aims. However, uptake of preventive and sexual health services and care by older men remains low at these facilities globally [12, 13]. Studies across different contexts, including geographic areas, delivery methods and age groups, provide different reasons to explain this occurrence. Some of the recurring factors associated with access to care by men and actual uptake of healthcare include costs of services, distance to facilities, cultural constructs of gender health needs, perceived quality of care and individual health goals [14–21]. Whereas it may seem obvious that the barriers are widespread, VMMC is fraught with complex socio-cultural and behavioral undertones which could impact on the uptake among older adults, which pose considerable challenges even with robust strategic interventions in place to address them. For example, while the program introduced free transport to address distance barriers as well as provision of inner-pants, peer counsellors and facility organization, among others, to address respective barriers, still there were no dramatic changes in demand, indicating probable influence of unapparent and seemingly more subtle issues. Furthermore, anecdotal field experience and program reports showed a differential or potentially selective responses. Whether these are global across all services or uniquely differ for VMMC is still understudied. The current national AIDS strategic framework has prioritized VMMC for expanded scale up alongside other high impact quality interventions [22]. Understanding factors that influence men’s choice regarding where to go for VMMC and sexual healthcare will guide interventions to improve scaled up service delivery, including strategic communication plans and framing promotion and prevention messages to increase demand and use of these services.

To foster wider community discussions about male circumcision and promote VMMC uptake, the Kenya national VMMC program adopted theory-based targeted health behavior change communication and promotion strategies [23, 24] focusing on segmented audiences according to their respective socio-cultural environments [19, 25, 26]. Psychological theories and models are important in guiding effective design of interventions to optimize desired outcomes; identify target behaviors for change and how to achieve the changes, and; conceptualizing implementation and evaluation plans. Implementing these strategies within the tenets of primary healthcare was expected to not only improve community knowledge, attitude and practices regarding sexual and reproductive health but also strengthen stakeholder engagement, participatory agenda setting and advocacy action plans envisaged in the ‘Kenya HIV Prevention Revolution Roadmap 2030’ [27]. The roadmap emphasizes the need for efficient delivery of combination prevention packages, synergistic integration of biomedical, behavioral and structural interventions. Primary healthcare facilities, which are the mainstay of universal healthcare, are ordinarily designed to provide basic care to the general population through an ‘all under one roof’ (integrated primary healthcare) approach. However, there is reported widespread disparities as well as lack of sexual and reproductive health service availability and readiness in Kenya. There is need for evidence to inform efforts to address the differing needs and constraints of males, particularly with regard to VMMC and sexual health-seeking [28]. This paper sought to understand the factors that influence uptake of VMMC by older men aged 25–39 as well as their preferences in choosing where to go for VMMC services in the context of primary healthcare.

### Conceptual framework for analyzing facility preferences

The study adopted a ‘patient-centred access to health care’ framework by Levesque et al., [28] to enable a comprehensive analysis of multi-level dimensions of supply and demand which influence access to and use of services along the care continuum. Using this framework, we viewed access as the ‘opportunity to identify male circumcision needs, make decisions to seek, reach, obtain or use services, and to actually have a need for services fulfilled’. Considerations of whether and where to attend VMMC services were deemed to be a function of multiple interacting elements of both supply and demand factors, such as facility location; availability, cost and appropriateness of services as well as perceived ability to seek, reach, and pay for services.

Key components of the conceptual framework used for analyzing facility preferences included the following: accessibility/approachability; acceptability; adequacy; affordability; and availability as well as role of context (resources, structures, institutions, procedures, and regulations through which health services are delivered) as proposed in Levesque et al., (2013), Jacobs et al., (2012) and Obrist et al., (2007) [29–31]. Table 1 summarizes the attributes of each component as applied in this analysis. From the perspective of health systems functions, these components also correspond to contextual social and service delivery factors that influence VMMC care delivered. These factors include facility infrastructure (physical structure: space, location of services; equipment and supplies; client load and flow; facility level by MoH category, and guidelines on service delivery); process (inputs involved in care delivery such as clinical care, orientation of care, interpersonal care, prioritization of needs, efficiency of care and organization) and outcome (effects, such as met needs). The underlying assumption to our analysis is that clients are rational and actively seek out information concerning VMMC health facilities or rely on previous experiences or information from friends/family to choose facilities where they can get high quality services while minimizing costs (usually direct and indirect) potential harm, or other adverse outcomes.

**Table 1:** Dimensions of access to health care services and their attributes

Dimensions and definitions	Components / factors
**Availability**: existing health services and products meet client needs	Services package and physical space available at usual sources of care; provider characteristics (e.g. skills correspond with services offered); equipment and supplies suffice to cover the demand.
**Geographic location** / **approachability**: The location of health service supply is in line with the location of clients.	The geographical distribution and distance between the facilities/services and the homes of the clients; means of transport to preferred service delivery point; average time to reach service delivery points.
**Affordability**: Direct / indirect cost burden.	The direct costs of the services; indirect costs in terms of transportation, lost work time and income.
**Adequacy**/ **appropriateness**: The organization and management of health care meets client expectations.	How services are organized and the specific elements that meet clients’ personal health and psycho-social expectations: service quality, communication, consultation schedules (e.g. opening / closing hours match with schedules of clients), timeliness, facilities are clean and well kept.
**Acceptability** & **motivations** Characteristics of providers and services correspond with expectations of the clients.	Health information and services provided consider local health concepts and socio-cultural values; client awareness of own health needs - vulnerabilities to health threats and severity of the health threats; perceptions and response actions; capacities to enable coping.

Adopted from Levesque et al., (2013), Jacobs et al., (2012) and Obrist et al., (2007) [28–30]

## Methodology

### Study setting

This qualitative study was conducted between May 2014 and June 2016 as part of a larger cluster randomized controlled trial *(NCT02497989)*. The parent study aimed to assess the impact of two demand creation interventions (interpersonal communication and dedicated service outlets) among adult men aged 25–39 years, which although is a priority age-group for the national VMMC program yet, consistently showed suboptimal service uptake. The demand creation interventions were delivered separately and together, on VMMC service uptake in former Nyanza province, western Kenya, an area that is predominantly occupied by a traditionally non-circumcising Luo ethnic community [32]. During the qualitative phase of the larger study, we conducted 24 focus group discussions (FGDs) and 48 in-depth interviews (IDI), in order to understand the factors that influence older men’s uptake of VMMC to better inform the larger RCT’s goals of demand creation for these services. The parent study adopted FGDs and IDIs Questionnaire guides comprising open-ended questions with appropriate probes where necessary. However, for this current study, only information from 6 FGDs with circumcised men and 6 FGDs with their partners were considered.

### Study population

We purposefully selected four distinct participant groups of circumcised men and their sex partners to take part in the qualitative component of the study. To be eligible, male participants had to be aged 25–39 years, resident within the locations excluded from but proximal to the main study Locations, have disclosed circumcision status to their partner, and given written consent to participate in the study and to be audio-recorded. Primary female partners (as identified by the male partner) were eligible if they resided within the study region, were ≥ 18 years old, were aware of their partner’s circumcision status, and gave written consent to participate in the study and to be audio-recorded.

### Study methods – tools and data collection

Qualitative study during the formative stage of the parent study adopted focused group discussion questionnaire guides included open-ended questions with appropriate probes where necessary. A total of 24 FGDs were conducted (12 with circumcised males and 12 with female partners). The number of participants was 6–10 per FGD. For the circumcised men, 12 FGDs conducted were spread proportionally across the southern and northern Nyanza regions (2 for each of the age categories: 25–29, 30–34, 35–39 years); similarly, 12 FGDs were conducted with female partners of the enrolled male participants.

Potential participants were mobilized by the VMMC program demand creation staff (community mobilizers) across the two geographical administrative locations adjacent to, but separate from, the respective clusters selected for the main study. To ensure satisfactory geographical spread, the community mobilizers were identified through simple randomly sampling and assigned to recruit 1–2 participants in the required age bracket and circumcision status. Potential participants were referred to study staff of the same gender at a pre-arranged venue where the study was explained, eligibility assessed, and consent obtained for participation. Study staff were fluent in English and the local language (*Dholuo*) and had been trained on the study protocol and relevant ethical issues. The FGD interview sessions were conducted in private, audiotaped and transcribed, with simultaneous translation into English, where necessary. Both FGDs with male and female participants explored the same issues. Each FGD lasted approximately 2 h. Discussion interview questions focused on the underlying personal preferences and other factors, particularly facility characteristics that influenced the decision of men to go for VMMC services. These factors included recruitment forums (social meeting places/listed study households), characteristics of the recruiter, gender of service provider at the clinic, staff-client relationship dynamics, and layout of the clinic. Also considered were men’s preferences for age and sex of various clinic staff by role (i.e., performing the physical exam, performing the surgery, assisting the surgeon, and conducting follow up reviews); preferences related to the day of the week and time services are offered, the location of services, and the presence of other VMMC clients of mixed ages; and whether requirement for HIV testing had an influence on decision for VMMC service uptake.

### Data analysis

The current study used a predetermined representative sample size for FGDs as estimated for the parent cluster randomized intervention study. The study had multiple end points for both qualitative and quantitative events and the sample size estimation catered for the complex design. A systematic review by Hennik and Kaiser [33] showed that ‘Studies using focus group discussions reached saturation within a narrow range of interviews (4–8) particularly those with relatively homogenous study populations and narrowly defined objectives’. In the current study we used all the 12 FGDs from the parent study representing distinct administrative regions of interest. These groups were independent and homogenous.

Qualitative data from the parent study had been transcribed, coded and analyzed through content analysis techniques by the study staff who had conducted the FGDs. Secondary analysis of these codes, developed from analysis of all FGDs transcripts from the parent study, was performed manually by concurrently reviewing outputs for males and their partners. To identify concepts and ideas relevant to the framework, text structures were explored to abstract underlying meanings, patterns, context and potential implications of the information in relation to the respective dimensions of the conceptual framework without regard to saturation level as the focus on identifying patterns, themes, structures and relationships within the data, rather than seeking exhaustive coverage. Identified items were fitted to the framework dimensions. While we did not estimate level of data saturation given we used a predetermined framework, we deemed the predetermined sample size used to be representative and adequate for the study.

Data triangulation was undertaken through comparing findings derived from different respondent groups, geographical locations and data sources (FGDs). This was to not only provide a comprehensive picture of participants’ VMMC-related perceptions and experiences in the study settings but also, ensure validity and reliability. Interpretation of the data text structures was summarized according to the predetermined themes as proposed in Levesque et al. [29] Obrist et al. [30] and Jacobs et al [31]. Illustrative quotes were identified to demonstrate prevailing perspectives, meaning, implications or contexts relevant to the study objectives. Using interpretive coding approach provided further insight about patterns and inference about potential relationships in the coded data. The framework adopted for analysis of health facility choices, preference and VMMC service use enabled simultaneous consideration of multifaceted perspectives on VMMC demand- and supply-side factors as elicited from circumcised men and their partners.

### Ethics statement

The parent study had been reviewed and approved by the University of Nairobi-Kenyatta National Hospital Ethics Review Committee *(P36/02/2013)* and the CDC Institutional Review Board *(6456)*. Written informed consent for IDI and FGDs with circumcised and uncircumcised men and their sex partners had been obtained at study enrolment. Before the start of interviews, the moderator emphasized to participants their voluntary participation and right to withdraw and leave at any time if unwilling to be audio-taped. To mitigate potential breach of confidentiality, each participant was given a pseudonym to use rather than their name throughout the session. All FGD participants were provided with refreshments, reimbursed for transportation up to a maximum of 200/-, and received a modest compensation at KSh. 250/- (below the current minimum wage in Kenya) for time taken to participate in the study. and modest compensation for time taken to participate in the discussion (at the time the USD exchange rate to the Kenya Shilling was 1USD = 80/-).

#### Considerations for preferring FGDs

Whereas in the study context, male circumcision may be regarded as a personal issue and individuals might not go public about it, we argue that male circumcision is also largely a socially acceptable procedure and is publicly discussed among peers. In the context of medical male circumcision programming, there is widespread and intense community campaigns to sensitize communities. The participants were free to interrogate perspectives from fellows and build consensus where need be. Holding FGDs ensured that particular viewpoints were confirmed or interrogated from different sources of knowledge/experiences. In addition, the focus group participants were as homogenous as possible to enable openness and trust to discuss common experiences. Only consenting individuals were recruited. Also, the circumstance of conducting FGDs ensured both confidentiality and privacy to mitigate unwarranted outcomes.

## Results

**Table:** 

Summary of themes: Conceptual dimensions	Thematic issues
Service Availability:	Smaller and lower level facilities are less preferable because they are more likely to lack competent service providers, offer low quality and a limited range of services, and; they lack essential equipment and space. Preference by facility location differed according to individual’s personal motivation or inclination to engage in sexual and reproductive health care.
Accessibility/approachability:	A health facility may be preferable to attend if the individuals (index male and/or sex partner) consider it of high repute and if it was perceived as being able to guarantee favourable social context and privacy, regardless of costs involved.
Affordability:	Flexible clinic schedules could offset disadvantages due to costs attributable to distance covered to facility and waiting time at the clinic.
Adequacy/appropriateness:	A facility considered to be ‘friendly’, if clean and well maintained, caters for privacy, operates flexible clinic schedule including odd hours throughout the week, offer skilful post-operative counselling, care, and referral.
Acceptability:	Discreet diagnosis and treatment of HIV and other STIs at a facility supports uptake of stigmatised services; Perceived health needs, perceived ability and self-efficacy to seek help to meet felt needs and health motivations;

### Service availability and facility infrastructure

Avoidance related to perceived stigma and social mixing was considered in choosing whether or when to visit a facility. Smaller facilities, like the dispensaries (the first level of skilled healthcare in Kenya) or health centers, were considered by some of the male and female participants as unlikely to provide adequate social boundaries (group distinctions based on social relationships or cultural status/role), confidential care and privacy. Public health facilities, most of which provided basic healthcare services and referrals, were perceived by participants to be primarily oriented to care for women and children, and staff less skilled in providing VMMC. However, concerns on maintaining social boundaries were still reported as a potential barrier even in situations where skilled staff were available. The facility infrastructure and physical lay-out in most of these facilities were perceived as unfriendly to men’s care.

Both females and males considered it culturally inappropriate for a man to attend circumcision services with adolescent boys because of a need to maintain social boundaries. In such contexts, men expressed reluctance to access health facilities closer to their residence. Whenever possible, men preferred to bypass closer health facilities and attend those further away in hopes of circumventing potential stigma and other social challenges. Even then, this had to be discreet. In addition, participants needed to be certain that the facility they opted to attend offered VMMC services, was ‘well equipped’ to perform circumcisions safely and, if needed, provide referral for complicated cases and treatment for sexually transmitted infections.

The requirement for HIV testing as part of VMMC services was identified as problematic for men but not all participants had the same idea. There were three negative aspects of HIV testing before circumcision. First, the fear of testing positive for HIV infection; second, the perceived potential of a positive test as being highly likely to rock relationships with peers, sexual partners or parents, and; third, the likelihood of individuals becoming fretful and despondent at personal levels. On the other hand, some of the female participants considered circumcision as well as HIV testing services are both important health services just like any other medical interventions, and should not be a problematic issue for men seeking VMMC services.

Service provider characteristics including age, marital status, how they interacted with patients, task competency and interpersonal communications were reported as important service access dimensions, but perspectives varied. There were concerns about poor provider–client interactions, their knowledge and, competence and experience with circumcision. For some, getting the services was paramount over other factors. While the age or sex of service providers was not necessarily a priority, biases were reported related to gender of circumcision team; social stereotypes (only male service providers should perform circumcision), provider maturity (older men should be circumcised by peer male service providers); perceived mannerisms (female staff might unduly massage the male genital at surgery); or individual ambivalence (it doesn’t matter who performs the circumcision). Table 2 identifies some of the quotes.

**Table 2:** Participants’ perceived influence of gender of male circumcision providers on access to services

Factors influencing access & preferences	Sample quotes
**Provider characteristics**: There were differing opinions among male and female participants in their preferences regarding gender of provider performing circumcision.	*… [a] man will feel embarrassed that a female is circumcising him. (FGD, female)* *some people see a difference … there are some men who only prefer the female while others prefer male providers. They differ. [FGD, female]* *… A female surgeon should not touch my penis (FGD, male)*

### Accessibility of services

Attendance at a given facility for VMMC was not straightforward. Factors influencing facility selection included physical access of the facility, travel cost and travel time involved. Males seeking VMMC and HIV testing services further from their usual residence was reported as a way to avoid friends, relatives or situations that might expose them to real or perceived embarrassment or ridicule despite extra costs and travel time. Both male and female participants noted that, even where transport was facilitated to reduce travel time and transport costs, certain individuals still avoided using them altogether or preferred to be picked or dropped a distance far from their homestead to avoid being ‘seen’ to have gone for circumcision (see Table 3).

**Table 3:** VMMC services access, decision-making and challenges

Factors influencing access and preference	Sample Quotes
**Facility location**: Preference for facilities located within one’s area of residence varied according to logistical practicalities, expediency and need to avoid being noticed by persons familiar to them.	*I feel that VMMC facilities should be very near to the people, so that … once they are sensitized, they can find nearby centers where they can straight away get the service. (FGD, male)* *Men … will accept to go to hospitals where services are being offered … if they are located far [from home] (FGD, female)* *Some of the people … fear to be seen by others that they went for VMMC, so … they prefer going far away from their village yet they don’t have the time and transport. (FGD, male)*

### Service affordability

Participants consistently identified forgone work time/wages and waiting time at the facility as a key issue to facility preference. However, widespread availability of free VMMC services and flexible schedules offered older men more access opportunities and motivation (Table 4).

**Table 4:** Service affordability, time burden and facility preferences

Factors influencing access & preference	Sample quotes
**Time as a cost**: prolonged travel and waiting time were challenges; Facilities that operate night or weekend shifts accorded clients flexible moments and favorable alternatives. Out-of-pocket costs associated with transportation.	… *The distance and the time spent at the circumcision clinic … is a lot … (FGD, male)* *… Men will use VMMC services [when they go] for the services for free … given lunch and some allowance that will sustain him even for three days after circumcision. (FGD, female)* *‘…most people would prefer that circumcision is done at night when very many people are not there … [and] more doctors should be available to reduce time wasting’. [FGD, male]* *Distance is a challenge because maybe someone has no fare to reach (take) him to the place. (FGD, male)*

### Service adequacy/appropriateness

Overall, participants consistently indicated preferences for facilities they perceived would honor and protect their dignity and values, particularly from both overt and subtle discrimination with regard to consultations for HIV & sexually transmitted infections (STI)-related and circumcision services. Both male and female partners expressed feelings of embarrassment related to taking up circumcision at an older age (mature males) as well as seeking treatment services for HIV/STI infections (services they associated with illicit sexual affairs); provider interpersonal and technical skills were considered important to ensure delivery of satisfactory services and provision of clear information about VMMC services. Whereas there were concerns among some men with components of VMMC, particularly HIV testing services (HTS) and deploying female clinicians as male circumcision surgeons and assistant surgeons, these were considered secondary to providing quality healthcare. Technical skills were perceived as essential to management of post-circumcision challenges such as infections and timing of resumption of sexual activity. Preferably, the facility needed to be ‘friendly, clean and well maintained, with flexible service hours daily throughout the week and ensures privacy’ (Table 5).

**Table 5:** Participants’ perceived facility service readiness and appropriateness for HIV & STI care

Factors influencing access and preference	Sample quotes
**Privacy**: It is a component in preserving personal dignity and social status since discrimination and embarrassment related to HIV and circumcision services are still pervasive. **Adequacy of counselling (by service providers)**: it should be discreet, clear and adequate to enable better pre- and post- circumcision decisions.	*… [Because of] fear that if he is seen going into the VCT (voluntary [HIV] counseling and testing) center, it may be concluded that he is sick (with HIV) and has gone to collect medicine. (FGD, female)* *To encourage men to go for VMMC, first the VCT should not be part of the VMMC process (FGD, male)* *… When you come from that place no one [should] know where you are from. You come from that place directly to your house that only you and your wife are the two people who know. (FGD, female)* … *[counselling] should not be done at a public place for many people to know what is[being] done [FGD, female]* *‘but if you go to a distant hospital [the service provider] welcomes you happily, … talks with you politely and explaining nicely to you about male circumcision (FGD, female)*

### Acceptability and motivations for care actions

The participants indicated that while HIV and sexually transmitted infections (STIs) are serious and sensitive health threats, VMMC service package offers opportunities for men to protect against these conditions. However, they acknowledged that circumcision decisions are complex because of socio-cultural nuances, particularly pertaining to gender roles, sexuality and the expected ‘sick-role’. The benefits of VMMC in protecting men and sexual partners from sexually transmitted diseases was acknowledged across groups. Furthermore, the participants indicated that decision-making and accessing the services had to be approached discretely and cautiously while dealing with personal and interpersonal social norms. Participants indicated that seeking VMMC services for prevention of HIV and other STIs and where to attend was challenging as these conditions were perceived to occur only among promiscuous people. Participants noted that navigating through all these complex issues was not a straight forward matter despite awareness and desirability of VMMC. This made many individuals hesitant in making the decisions. ‘Circumcision is not part of *Luo* culture’ was often cited as a cause for older men declining VMMC even with the option of choosing facilities located well away from their residence or offers for transport. Some of the participants indicated that female partners or peers who embraced VMMC play critical role in persuading or supporting males through the decision making, service seeking and post-circumcision processes. In addition, the individual’s intentions, and uptake of VMMC services were influenced by personal factors, including one’s health motivations, self-efficacy, resolve to be circumcised, knowledge of alternative VMMC care options and where to go for these services. Participants widely recognized that the challenges men face with actual uptake of circumcision can be resolved through discussion with female partners, peers or by service providers. Some female participants noted that health services are universally provided to everyone, and men should not have reservations or fears because service providers are always there to ‘help’ them (Table 6).

**Table 6:** Social norms complicate individuals’ decisions to engage with VMMC services

Factors influencing access and preference	Sample quotes
**Acceptability**: the personal drive to engage with VMMC services for its health benefits tends to conflict with inherent expectation to ascribe to normative ethnic standards, with differing results	*[Previously] circumcision [status] was something which if you revealed amongst the people it was taken as if you are coming from another tribe. And after it was rolled out, I decided not be left behind … I had a problem before roll out … I didn’t want to be different from my people. (FGD, male)* *‘Some people are ashamed of others knowing that they are circumcised, and so they hide it. Also, some people consider those who are circumcised to be outcasts, so they will boo or sneer at you, when you are bathing at the lake [FGD, male]* … *if you want assistance you should not [be choosy] [FGD, female]*

## Discussion

The study findings showed that older men’s choices and preferences of facility to attend for VMMC services are influenced by diverse and complex factors cutting across individual-, interpersonal-, facility-, and community-levels. Men’s decisions regarding accessing and usage of healthcare services for VMMC are embedded in their broader health goals, personal experiences and their life situations [34–36]. These included individual-level characteristics such as one’s health attitudes and values, knowledge of VMMC services and ability to balance health needs versus desire to conform to social-cultural demands; health facility characteristics including location, physical layout, organization of patient flow, infrastructure and service provider skills related to VMMC and STI services; and social and cultural context (including family, culture and peers). Whereas the challenges expressed by men and their female partners participating in this study on use of VMMC services are context based, they provide insights on the scope of issues contributing to sub-optimal demand for VMMC services in the VMMC priority counties.

### Service availability and facility infrastructure

Service availability, organizational and facility infrastructure in Kenya are largely based on standard guidelines by the according to the Ministry of Health designated service level yet, operational characteristics may vary. Individuals tend to evaluate mentally the relative attractiveness of the facility characteristics in order to choose to attend or not [17, 37]. The perceptions about VMMC, as shaped by cultural beliefs, economic considerations, and personal experiences, may interact in unique ways to influence choice and preference of the facility to attend, if at all [24] and [25]. In the current study, the reasons for facility preference decisions were varied among participants, ranging from nudges from multiple diverse sources, to individual perceptions including characteristics about the provider, waiting time, facility layout/infrastructure and spatial location. In addition, subtle gendered differences were observed in participants’ choices to seek VMMC services and preferences of facility. This signifies the importance of gender roles in motivating or hindering choices and preferences in healthcare related behavior. Whereas male participants considered and sought for ‘safe spaces’ to receive circumcision services, this selective preference of which facility to attend for VMMC services was deemed by a female partner to being ‘choosy’ of an essential health intervention.

In the context of VMMC service delivery to prevent HIV and other STI, VMMC provides a platform for males to access essential sexual and reproductive health services. However, intersecting issues interacting with an individual’s health functions in the context of programming for HIV/VMMC may pose challenges to health seeking and increase vulnerabilities for both men and their sex partners to poorer outcomes. Both conditions are priority health conditions yet, were observed to be fraught with multiple psychosocial and sexual connotations. Ensuring availability of the comprehensive VMMC service package is needed to not only address social-level factors intersecting with sexuality and gendered decision making but also, promote access to other sexual and reproductive health services. Understanding intersectionality is specifically helpful in developing responsive and targeted person-centered intervention components [37]. Previous studies indicate that availability of intervention packages for VMMC may need to simultaneously target cross-level factors, such as, building capacity of service providers (supply-side strategy), while reducing the access and participation barriers coupled with efforts to promote positive male health-seeking behavior change (demand-side strategy) [31, 38].

### Accessibility/approachability

The physical location, means of travel and ability to reach the facility (geographical accessibility) elicited mixed perspectives, depending on the individuals’ mundane life situation, motivations and health seeking intentions and/or priorities. For example, some individuals would overlook the cost factor if they prioritized privacy or quality of care. On the other hand, patient flow and time schedules were considered important. In Kenya, the public healthcare facilities are structured hierarchically and designated from lowest community health unit (Level 1) to specialist referral hospitals (Level 6) [39]. There were concerns from some male group participants that primary care facilities were designed to cater mainly for females and children (where children in the study context include teens). In contrast, female participants’ argued that services were gender neutral. This highlights possibility that heterogenous groupings might exist in the population each with distinct social characteristics. Of the essential health programs provided at the facilities, maternal and child health service programming has been purposefully tailored to meet unique health needs of mothers and children. Adopting best practice strategies from similar program could help improve VMMC programs.

### Affordability

Spatial location of service delivery point is an important indicator of the geographical access to VMMC services. During the scale up of VMMC services, the program adopted mobile and outreach services including post-circumcision follow up care to mitigate physical and time barriers to accessing the services [8] and [9]. However, in the sustainability phase, VMMC services are offered through integrated service delivery at the primary healthcare facilities. The primary health care networks (PCNs) is expected to play a crucial role in the strengthening VMMC service delivery, their main objectives being to improve access, deliver cost-effective services, enhance the quality of care, and contribute to research and innovation. Expanding access through working with care providers at the established care networks and strengthening the referral systems to ensure that patients receive appropriate care at the right time, could help achieve universal access for VMMC. However, the impact expected may vary according to the relative location, characteristics and organizational infrastructure of health facilities providing VMMC services. In the current study, the impact of facility characteristics and geographical location on preference for nearest facilities varied across differing factors, including situational expediencies, logistical practicalities, and need to conceal sexual and reproductive health status or needs. The evidence of multiple shades of opinions on why the individuals preferred the nearest or further-away located facilities indicate either changing priorities or unmet needs. Hence, program managers could advocate for systems strengthening through responsive interventions [17, 37].

### Service adequacy/appropriateness

Some aspects of the recommended VMMC program components and strategies were not considered favorably. Promotion of HIV testing services (HTS) alongside VMMC, deploying female clinicians as male circumcision surgeons and assistant surgeons, facilitating transport to and from facility and setting up VMMC service units in a section open to public view, were considered undesirable by some. Conversely, others indicated that VMMC service components as offered were necessary to meet their health needs. This signified that either the services as implemented were not meeting desired needs or a tacit dissatisfaction with aspects of its delivery or declension of these service components altogether. However, it might also imply, as in other African regions, that individuals considered it unwarranted if they perceived they are not at risk of HIV infection, fearful of possible positive test outcome, not guaranteed reasonable privacy and confidentiality, and apprehensive of HIV-related stigma [40, 41]. Hesitancy for HIV testing during VMMC service provision among older men in this population may be partly attributable to structural, facility lay out and organization. Concerns for privacy, clarity and adequacy of counselling indicate needs to be met at the facility-level. These challenges may require additional policy and targeted administrative interventions to address [31, 41].

### Acceptability and motivations for care actions

The benefits of VMMC in protecting men and sexual partners from sexually transmitted diseases was acknowledged across groups as a key motivation for seeking VMMC services. On the other hand, participants acknowledged that circumcision decisions remain complex because of socio-cultural nuances, particularly pertaining to gender roles, sexuality and the expected ‘sick-role’ in the immediate post-operative period, when physical or social activities may be limited by experience of pain. Also, participants noted that promoting circumcision as prevention of HIV and other STIs implied it was only for promiscuous people. Similarly, Plotkin et al., [42] in a VMMC program scale up context showed that, male participants expressed concerns with sharing clinic spaces with younger men, which may reflect normative beliefs around *‘who’* medical circumcision is for. Because of the stigmatized HIV and VMMC contexts, ordinary primary care facilities were perceived to be limited in providing satisfactory care for these conditions among older males.

These findings have important implications for delivery of health promotion and education services with regard to target setting and the content of the information and communication package. Understanding and responding to the inner desires and motivations factors that drive choices and preferences for services and delivery points among target audience could enable further improvement in communication activities. Messaging and message framing approaches targeting key motivation and decision drivers could strengthen acceptability and social desirability of medical circumcision as a health promoting intervention. Improvement interventions could be designed to simultaneously enhance acceptability of VMMC service package components in the population; reinforce acceptability of VMMC as a felt need to for its health benefits; enable informed sexual health choices, and; strengthen nudges to the personal drive to engage with VMMC services against conflicting ones. Additional research to identify these factors is needful for improved VMMC programs delivery among the target group.

These observations from the current study paint a complex and nuanced picture of VMMC service choices and preferences in Kenya. Previous studies on healthcare decision making [14, 19, 36, 43]– [45] showed that individuals’ choice and preference for health facilities was multifaceted and heterogenous. It does not necessarily depend on availability and location of safe, high-quality care, low cost or affordable services, even though these qualities are beneficial. Also, utility and preferences of ‘user friendly’ units believed to best fit community’s needs may vary over time as one’s psychosocial inclinations, health context and health status change. There is no ‘one size fits all’ implementation approach for all settings and service components. Further research to establish interaction among various access and usage components, including service availability factors, personal and interpersonal variables that cause access variability is needful to guide resource planning and responsive service delivery.

#### Study limitations

We recognize that VMMC services are multifaceted, and a comprehensive analysis of intersecting gender and health systems issues was not feasible in this study. This may preclude generalizability outside the study context. Aspects of male circumcision services are apparently more complex than addressed in current implementation guidelines. Interpretations may vary across social and health service contexts of individuals observed. Despite these, sampling widely and inclusion of both men and their female partners from across the study area provided a broad inclusion of perspectives and experiences. Additionally, the study was conducted early in the scale-up phase of the VMMC program, when the entire community was experiencing cultural shifting with surfacing new value structuring and patterns as far as male circumcision was concerned. Similarly, the health systems functions, particularly service delivery management, communications and policy guidelines for implementation of VMMC service package were still in their nascent stages. Hence, participants’ contextual understanding, and individual perspectives of different aspects of VMMC service package were likely still evolving. However, the thematic issues elicited appeared coherent and reveals what might be expected during scale up of a new or complex health service or technologies among naïve communities previously inexperienced with it.

## Conclusion and key considerations

Factors underlying preferences of older men (aged 25–29; 30–34; 35–39 years) for selection of facilities to attend for VMMC services are diverse, involving the interplay of individual, structural and socio-cultural considerations. Interventions that simultaneously address the different service access barriers to VMMC for older men in this community increases the likelihood that individuals who are aware and motivated will access and use the services consistently. Additionally, teams providing VMMC services may want to explore behavioral nuances unique to their operational contexts and tailor service delivery to address unique sub-groups.

Future studies regarding older men’s decision making and participation in VMMC for HIV prevention services could provide additional evidence to inform messaging and facility improvement plans. Programs may enhance engagement of female partners of older men not only as key influencers of male health seeking but also, as potential secondary beneficiaries to male circumcision through improved health outcomes. Additionally, monitoring and evaluation metrics are needful that capture access and use barriers more comprehensively. Policy makers, program managers and implementors should strengthen person-centered service delivery in the context of primary healthcare to ensure high quality care tailored to cater for individual context. This could help to reduce inequities in access, use and outcomes of medical male circumcisions and HIV programming.

## Data Availability

The datasets analysed during the current study are available from ClinicalTrials.gov/NCT02497989 repository or from the authors upon reasonable request and with permission of Impact Research and Development Information (IRDO) and CDC.

## Declarations

### Analysis and interpretation of data

Omondi Dickens Aduda; Spala Ohaga, Jacob Odhiambo, Kawango Agot.

### Wrote the paper

Omondi Dickens Aduda; Kawango Agot; Spala Ohaga; Jacob Odhiambo; Elijah Odoyo-June; Cathy Toroitich-Ruto CDC (qual.), Appolonia Aoko (qual.), Caroline Kambona (qual.).

### PEPFAR attribution of support

This publication has been supported by the President’s Emergency Plan for AIDS Relief (PEPFAR) through the Centers for Disease Control and Prevention (CDC) under the terms of 1U2GGH001226.

## References

[1] Joint United Nations Programme on HIV/AIDS (UNAIDS) (2021). Confronting inequalities: lessons for pandemic responses from 40 years of AIDS. Report No.: UNAIDS/JC3020E. Global AIDS update.

[2] Risher T, Cori KA, Reniers A, Marston G, Calvert M, Crampin C, Dadirai A, Dube T, Gregson A, Herbst S, Lutalo (2021). Age patterns of HIV incidence in eastern and southern Africa: a modelling analysis of observational population. Lancet HIV.

[3] Karim C, Baxter (2021). HIV incidence trends in Africa: young women at highest risk. Lancet HIV.

[4] WHO and UNAIDS. New Data on Male Circumcision and HIV Prevention: Policy and Programme Implications, *WHO Press*, no. March, pp. 1–10, 2007.

[5] Ministry of Health, National AIDS, Control Programme STI. Kenya AIDS indicator survey 2007: final report., 2009.

[6] Ministry of Health, Kenya. The Kenya Demographic and Health Survey (KDHS). p. KDHS 2014, 2014.

[7] Kenya National Bureau of Statistics. ICF Macro. Kenya demographic and health survey 2008–09. Calverton (Maryland): ICF Macro; 2010., Nairobi, Kenya, 2010.

[8] Hankins E, Warren C, Njeuhmeli C (2016). Voluntary Medical Male Circumcision for HIV Prevention: New Mathematical models for Strategic demand creation prioritizing subpopulations by Age and Geography. PLoS ONE.

[9] Government of Kenya. Ministry of Health National AIDS and STI Control Program. Natl Voluntary Med Male Circumcision Strategy, 2014/15–2018/19 Nairobi, Kenya. 2014.

[10] Kripke E, Reed K, Hankins J, Smiley C, Laube G, Njeuhmeli (2016). Correction: impact and cost of scaling up Voluntary Medical Male Circumcision for HIV Prevention in the context of the New 90-90-90 HIV treatment targets. PLoS ONE.

[11] Katharine Kripke M, Opuni M, Schnure S, Sgaier D, Castor J, Reed E, Njeuhmeli J, Stover (2017). Correction: age targeting of voluntary medical male circumcision programs using the decision makers’ Program Planning Toolkit (DMPPT) 2.0. PLoS ONE.

[12] Dowden Y, Mushamiri J, McFeely I, Apat E, Sacks D, Amor B (2019). The impact of ‘male clinics’ on health-seeking behaviors of adult men in rural Kenya. PLoS ONE.

[13] Beia K, Kielmann T, K., Diaconu. Changing men or changing health systems? A scoping review of interventions, services and programmes targeting men’s health in sub-saharan Africa. Int J Equity Health, vol. 201–16, 1, pp. 1–16, 2021. 10.1186/s12939-021-01428-z PMC8011198 33789688

[14] Victoor A, Delnoij DM, Friele RD, Rademakers JJ (2012). Determinants of patient choice of healthcare providers: a scoping review. BMC Health Serv Res.

[15] Musheke S, Ntalasha M, Gari H, Mckenzie S, Bond O, Martin-Hilber V, Merten (2013). A systematic review of qualitative findings on factors enabling and deterring uptake of HIV testing in Sub-saharan Africa. BMC Public Heal.

[16] de Arruda GO, Marcon SS. Survey on the use of health services by adult men: prevalence rates and associated factors. Rev Lat Am Enfermagem, 24, 2016. 10.1590/1518-8345.0296.2685 PMC4809176 27027680

[17] Hlongwa K, Mashamba-Thompson M, Makhunga T, Hlongwana (2020). Barriers to HIV testing uptake among men in sub-saharan Africa: a scoping review. Afr J AIDS Res.

[18] Galbraith JS (2014). Status of Voluntary Medical Male Circumcision in Kenya: findings from 2 nationally representative surveys in Kenya, 2007 and 2012. J Acquir Immune Defic Synd.

[19] Macintyre K (2014). Attitudes, perceptions and potential uptake of male circumcision among older men in Turkana County, Kenya using qualitative methods. PLoS ONE.

[20] Nxumalo GG, C. T., Mchunu. The development of an explanatory model for voluntary medical male circumcision in KwaZulu-Natal, South Africa. South Afr Fam Pract, 63, 1, 2021. 10.4102/safp.v63i1.5346 PMC8661273 34797095

[21] *Lancet*, vol. 393, no. 10189, pp. 2455–2468, 2019. 10.1016/S0140-6736(19)30765-2 31155273

[22] National AIDS Control Council (NACC). Kenya AIDS strategic framework II, 2020/21, 2024/25. Minist Heal Kenya, p. 80, 2020.

[23] Clearinghouse on Male Circumcision for HIV Prevention [Internet]. New York: AIDS Vaccine Advocacy Coalition; c2015., Eastern and Southern Africa Regional Meeting on Demand Creation for Voluntary Medical Male Circumcision, Lusaka, Zambia, April 3–5, 2013. [Online]. Available: http://www.malecircumcision.org/communication/Zambia_Regional_Meeting_April2013.html. [Accessed: 26-Dec-2021].

[24] Organization WH. A framework for voluntary medical male cirucumcision, 2016.

[25] Ackers M, Hightower A, Obor D, Ofware P, Ngere L, Kubaje A, Laserson K (2014). Health care utilization and access to human immunodeficiency virus (HIV) testing and care and treatment services in a rural area with high HIV prevalence, Nyanza Province, Kenya, 2007. Am J Trop Med Hyg.

[26] Golub G, Herman-Roloff A, Hoffman S, Jaoko W, Bailey RC (2016). The Relationship between Distance and post-operative visit attendance following Medical Male Circumcision in Nyanza Province, Kenya. AIDS Behav.

[27] Kenya MoH. Kenya HIV Prevention Revolution Road Map., 2014.

[28] Dworkin CJ, Fleming SL, Colvin (2015). The promises and limitations of gender-transformative health programming with men: critical reflections from the field. Cult Heal Sex.

[29] Levesque J-F, Harris MF, Russell G (2013). Patient-centred access to health care: conceptualising access at the interface of health systems and populations. Int J Equity Health.

[30] Obrist B (2007). Access to Health Care in contexts of Livelihood Insecurity: a Framework for Analysis and Action. PLoS Med.

[31] Jacobs B, Ir P, Bigdeli M, Annear PL, Van Damme W (2012). Addressing access barriers to health services: an analytical framework for selectingappropriate interventions in low-income Asian countries. Health Policy Plan.

[32] Odoyo-june E et al. Predictors of voluntary medical male circumcision prevalence among men aged 25–39 years in Nyanza region, Kenya : results from the baseline survey of the TASCO study, 851, pp. 1–10, 2017. 10.1371/journal.pone.0185872 PMC5628861 28982175

[33] Hennink M, Kaiser BN (2022). Sample sizes for saturation in qualitative research: a systematic review of empirical tests. Soc Sci Med.

[34] Wambura M et al. Increasing voluntary medical male circumcision uptake among adult men in Tanzania, *Aids*, vol. 0, no. Vmmc, p. 1, 2017. 10.1097/QAD.0000000000001440 PMC5378002 28350578

[35] Bronchetti E, Huffman ET, Magenheim (2015). Attention, intentions, and follow-through in preventive health behavior: field experimental evidence on flu vaccination. J Econ Behav Organ.

[36] *Lancet HIV*, vol. 2, no. 5, pp. e181–e189, 2015.

[37] *AIDS Care*, vol. 28, no. Sup 3, pp. 67–73., 2016.

[38] *Lancet (London, England)*, vol. 393, no. 10190, pp. 2535–2549, 2019. 10.1016/S0140-6736(19)30648-8 PMC7233290 31155270

[39] WHO. Primary Health Care Systems (PRIMASYS): Case study from Kenya, abridged version. World Heal Organ, pp. 1–16, 2017.

[40] Musheke M (2013). A systematic review of qualitative findings on factors enabling and deterring uptake of HIV testing in Sub-saharan Africa. BMC Public Health.

[41] Sanga Z, Kapanda G, Msuya S, Mwangi R (2015). Factors influencing the uptake of Voluntary HIV Counseling and Testing among secondary school students in Arusha City, Tanzania: a cross sectional study. BMC Public Health.

[42] Plotkin M (2013). Man, what took you so long?’ Social and individual factors affecting adult attendance at voluntary medical male circumcision services in Tanzania. Glob Heal Sci Pract.

[43] Abubakar A, Van Baar A, Fischer R, Bomu G, Gona JK, Newton CR. Socio-cultural determinants of health-seeking behaviour on the Kenyan coast: a qualitative study. PLoS ONE, 8, 11, 2013. 10.1371/journal.pone.0071998 PMC3832523 24260094

[44] Gasasira RA (2012). Determinants of circumcision and willingness to be circumcised by Rwandan men, 2010. BMC Public Health.

[45] Aduda N, Mkhize DSO (2014). Ethical issues evolving from patients’ perspectives on compulsory screening for syphilis and voluntary screening for cervical cancer in Kenya. BMC Med Ethics.

